# Chinese expert consensus on the management of aneurysmal subarachnoid hemorrhage-related hydrocephalus

**DOI:** 10.1186/s41016-022-00314-z

**Published:** 2023-03-20

**Authors:** Jun Pu, Yuan-li Zhao, Yu-xiang Gu, Chun-hua Hang, Yong‑ping You, Mao-de Wang, Yan Qu, Hua Lu, Shuo Wang, Chinese Neurosurgical Society

**Affiliations:** 1 Department of Neurosurgery, The Second Affiliated Hospital of Kunming Medical University, Kunming, China; 2 Department of Neurosurgery, Beijing Tiantan Hospital, Capital Medical University, Beijing, China; 3 Huashan Hospital, Affiliated to Fudan University, Shanghai, China; 4 Nanjing Drum Tower Hospital, Nanjing University Medical College, Nanjing, China; 5 Department of Interventional Neuroradiology, Jiangsu Province Hospital, Nanjing, China; 6 First Affiliated Hospital of Xi’an Jiaotong University, Xi’an, China; 7 Tangdu Hospital, Air Force Military Medical University, Xi’an, China

## Overview

Aneurysmal subarachnoid hemorrhage-related hydrocephalus (aSAH-H) refers  to a clinical syndrome of excessive secretion, obstructive absorption, or circulatory disturbance of cerebrospinal fluid (CSF) with subarachnoid hemorrhage following rupture of aneurysm that leads to excessive accumulation of intracranial CSF and enlargement of ventricles impairing neurological function. aSAH is an important risk factor for hydrocephalus [1, 2]. According to fluid dynamics, aSAH-H is divided into communicating and obstructive hydrocephalus; according to the time of occurrence, it is divided into acute (≤ 3 days) and chronic hydrocephalus (> 3 days) [3]; according to the progress of the disease, it is divided into progressive and arrested hydrocephalus; and according to intracranial pressure, it is divided into high pressure and normal pressure hydrocephalus. The incidence rate of acute hydrocephalus following subarachnoid hemorrhage by aneurysm is about 15–87%, mainly obstructive hydrocephalus, and a few patient falls into communicating hydrocephalus; the incidence rate of chronic hydrocephalus is about 8.9–48%, mostly communicating hydrocephalus [4–6].

Hydrocephalus often leads to cognitive impairment and neurological damage which hinder recovery. Moreover, intracranial hypertension caused by acute hydrocephalus may induce fatal cerebral hernia [7]. Chronic hydrocephalus causes symptoms such as cognitive impairment, incontinence, and gait disturbance, which adversely affects the quality of life for rehabilitation [2, 8].

Pathophysiology of aSAH-H is complex, and there is no standard diagnostic criteria and treatment protocol in China and abroad currently; there is also no consensus among clinicians on whether active treatment is needed and what treatment is preferred on aSAH-H. Therefore, diagnosis and treatment of aSAH-H have been puzzled by the unclarity and inconsistency of standards. To address these problems, Professor Wang Shuo, Chairman of the 8th Chinese Society of Neurosurgery of Chinese Medical Association, leads an expert group in China to compose this consensus with reference to many updated literatures home and abroad.

## Pathogenesis of aSAH-H

For aSAH-H, the main cause of obstructive hydrocephalus is blockage of pathway of CSF such as interventricular foramen, aqueduct of sylvius, and the third and/or fourth ventricle, and the main cause of communicating hydrocephalus is the absorption disorder and secretion increase of CSF [9]. Subarachnoid space filled by erythrocyte lysates, iron, etc. and blocked arachnoid granulation are the important causes of CSF absorption disorder [2]. Inflammatory response, disruption to the blood–brain barrier, etc. played a role in the fibrosis of arachnoid granule, making it dysfunctional and preventing the reabsorption of CSF [10–12]. Some recent articles also pointed out that neuroinflammation can promote the secretion of CSF and involved in the formation of hydrocephalus [13]. The coexistence of these factors contributed to the occurrence of aSAH-H [14]. In conclusion, increased production, decreased absorption, and circulatory disturbance of CSF are important pathogenesis of aSAH-H.

## Risk factors of aSAH-H

There are many factors related to the occurrence of aSAH-H. Age, location of aneurysm, neurological status onset, intraventricular hemorrhage, and volume of subarachnoid hemorrhage are closely related to shunt-dependent hydrocephalus after aSAH [1, 15].

Large volume of subarachnoid hemorrhage is an independent risk factor for hydrocephalus, and hydrocephalus is positively correlated with Fisher scale [8, 15, 16]. On one hand, the higher the score of Fisher scale, the larger the volume of hemorrhage it indicates in the ventricles and (or) the cisterns and also more obstructive for the circulation of CSF in the acute phase; on the other hand, the higher the score of Fisher scale, the more extensive aSAH is indicated and more degree of fibrosis in subarachnoid and arachnoid granulation in the late stage of aSAH [8, 16].

Intraventricular hematoma can affect CSF circulatory pathway in the ventricles, leading to acute obstructive hydrocephalus. Some scholars believe that intraventricular hematoma is a decisive factor for acute hydrocephalus, and it is also one of the risk factors for chronic hydrocephalus [15, 17, 18].

The location and size of aneurysms are also related to the occurrence of hydrocephalus, and they also affect the treatment of hydrocephalus in the acute phase. The incidence of acute and chronic hydrocephalus caused by aneurysm rupture in vertebrobasilar artery system is higher than that of in internal carotid artery system [15, 17]. In addition, previous literature reports that the bleeding times, cerebral vasospasm in the acute phase of subarachnoid hemorrhage, cerebral edema, and cerebral infarction are all related to the occurrence of acute hydrocephalus [15].

## Clinical manifestations of aSAH-H

The enlarged ventricular system suffers from hypoxia, ischemia, mechanical compression, and stimulation that lead to impairment of cerebral white matter. This mechanism reflects the clinical manifestations of hydrocephalus [19–21]. Acute hydrocephalus leads to CSF circulation and absorption dysfunctions, causing increased intracranial pressure (ICP), which leads to a series of clinical symptoms such as headache, projectile vomiting, papilloedema, and disorders of consciousness.

The progressive brain dysfunction of compressed brain tissue by chronic hydrocephalus is mainly manifested as the triad of cognitive dysfunction, gait disorder, and urinary incontinence. Among them, gait disorder is the most common, and about half of patients have the triad at the same time. Gait disorder is often the first manifestation. The mild cases are out of balance, and the severe patients cannot stand or walk. The typical manifestations are slow walking, unsteady swinging, short step length, increased step width, reduced foot lifting height, start-up, and turning difficulty, but arm swing is normal. Cognitive dysfunction involves all aspects of cognition, emotion, and mental behavior. Intelligence change appears early and often deteriorates within a few weeks or months. It starts with difficulty recalling information, then psychomotor retardation. In severe case, salient slow speech, reticence, degeneration of extremity movement, and its manifestations may be fluctuant or progressively aggravated in short term. Urinary incontinence is mostly caused by neurogenic bladder dysfunction with overactive detrusor function, which is generally late-stage manifestation, and there is seldom fecal incontinence.

## Diagnosis and differentiation of aSAH-H

The diagnosis of aSAH-H looks at medical history, clinical manifestation, image examination, lumbar puncture, etc.

1. History: A clear history of aSAH
2. Clinical manifestation
  a Headache, vomiting, and disturbance of consciousness are the main features of acute hydrocephalus.
  b Patients with chronic hydrocephalus may have one or more than one of the typical triad: Cognitive dysfunction, gait disorder, and urinary incontinence, but gait disorder must be included.
3. Image examination: Head CT is the most important and commonly used image examination to detect aSAH-H.

**Fig. 1:**
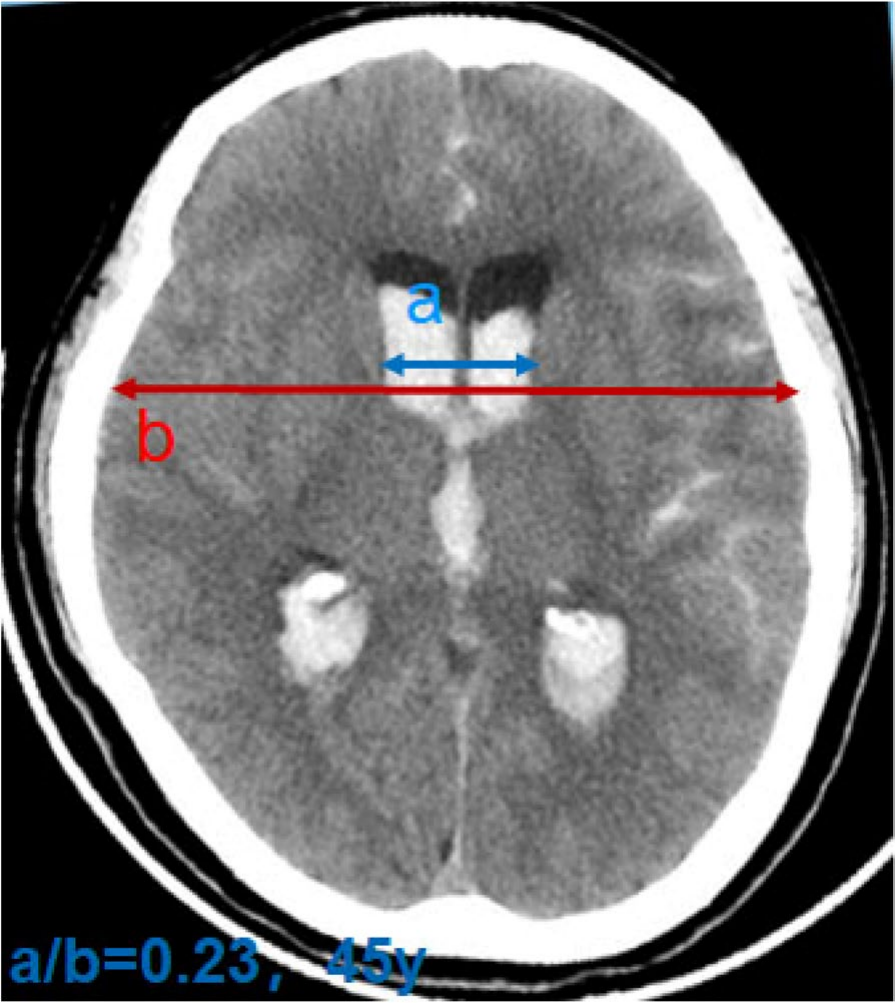
Acute hydrocephalus, bicaudate index (a/b)

**Fig. 2:**
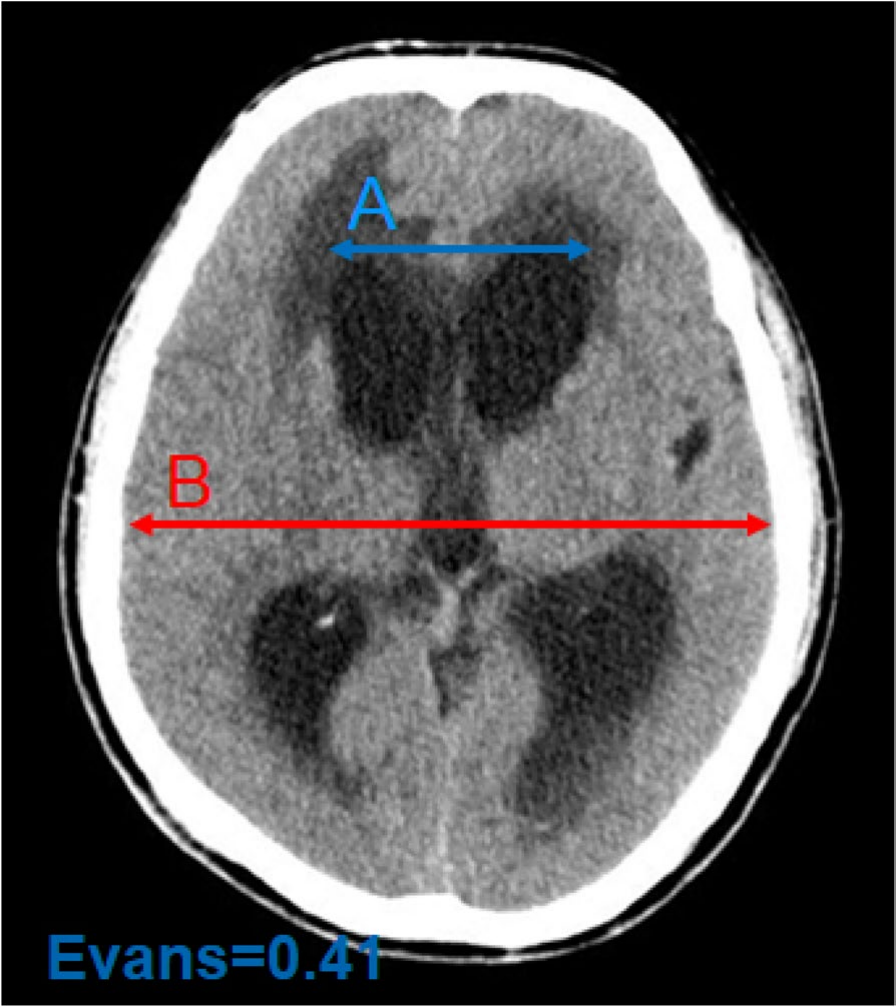
Chronic hydrocephalus, Evans index (A/B)

The typical manifestation is the abnormal disproportionate enlargement of ventricles, and there is no other cause for the enlargement. The bicaudate index (the ratio of the width of both lateral ventricles at the level of the heads of the caudate nuclei to the distance between the outer tables of the skull at the same level, a/b) exceeded the upper limit of 95% of the same age is taken as the diagnostic criteria for acute hydrocephalus (Fig. 1) [3]. The upper limit of 95% of bicaudate index for different ages is as follows: < 36 years = 0.16, 36 to 45 years = 0.17, 46 to 55 years = 0.18, 56 to 65 years = 0.19, 66 to 75 years = 0.20, 76 to 85 years = 0.21, and 86 ~ 100 years = 0.25 [3, 22]. Head CT shows enlarged bilateral lateral ventricles, interstitial edema around, round and blunt third ventricle; the ratio of maximum width of the frontal horns of the lateral ventricles, and the maximal internal diameter of the skull at the same level employed in axial CT (Evans’ index, A/B) > 0.3 is a typical marker for the diagnosis of hydrocephalus (Fig. 2) [23, 24]. For acute hydrocephalus, CT examination of the head shows brain swelling and disappearance of sulci, but manifestations like enlarged ventricular system and interstitial edema may not be as obvious as that of chronic hydrocephalus. In addition, some useful information of the white matter changes and the expansion of each ventricle from a 3D perspective could be provided by magnetic resonance imaging scan.

Typical clinical manifestation and image test can help make diagnosis basically, but differentiation of chronic hydrocephalus from delayed brain injury caused by subarachnoid hemorrhage, secondary ischemic injury caused by surgical clipping or endovascular coiling, as well as cerebral atrophy, vascular dementia, Alzheimer’s disease. Cerebral atrophy without other complications manifests enlarged ventricular system, with no interstitial edema and obscured sulcus; also, the patient will not demonstrate dementia, disturbance of consciousness, etc., and the clinical symptoms will not deteriorate. The diagnostic work-up of vascular dementia and Alzheimer’s disease is mainly based on image tests, and typical CT findings is beneficial to differentiation; lumbar puncture (TAP test) can ascertain the diagnosis of communicating hydrocephalus, while lumbar puncture is prohibited for obstructive hydrocephalus.

### Recommendations

If patient with aSAH has worsening disturbance of consciousness and deterioration of neurological function, after ruled out increased bleeding, the possibility of aSAH-H should be considered; head CT is the most commonly used examination method, and important indicators are abnormal enlargement of ventricles, bicaudate index, and Evans index for hydrocephalus; lumbar puncture is conductive to further clarify hydrocephalus.

## Progression of aSAH-H

**Fig. 3:**
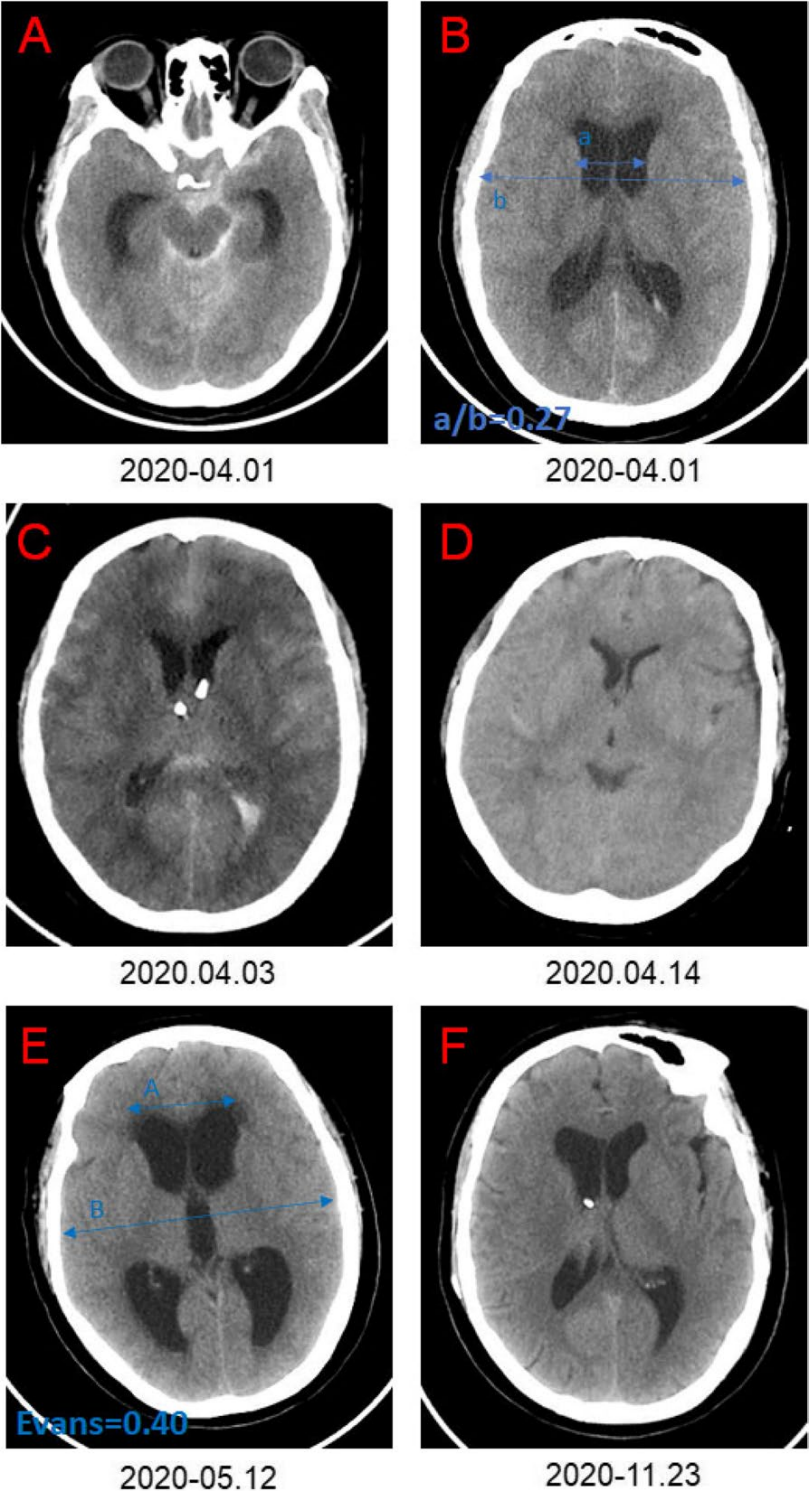
Progression of aSAH-H. **A**–**B** indicates subarachnoid hemorrhage complicated with acute hydrocephalus. **C** Bilateral external ventricular drainage. **D** Postoperative bilateral external ventricular drainage. **E** CT indicates chronic hydrocephalus. **F** Postoperative ventriculoperitoneal shunt

After aSAH, acute obstructive hydrocephalus may occur in the early stage. Symptoms may be relieved after a period of treatment. However, symptoms of chronic communicating hydrocephalus may appear gradually in 2–3 weeks, due to fibrosis in the subarachnoid space and cerebrospinal fluid absorption disorder (Fig. 3).

## Treatment of aSAH-H

**Fig. 4:**
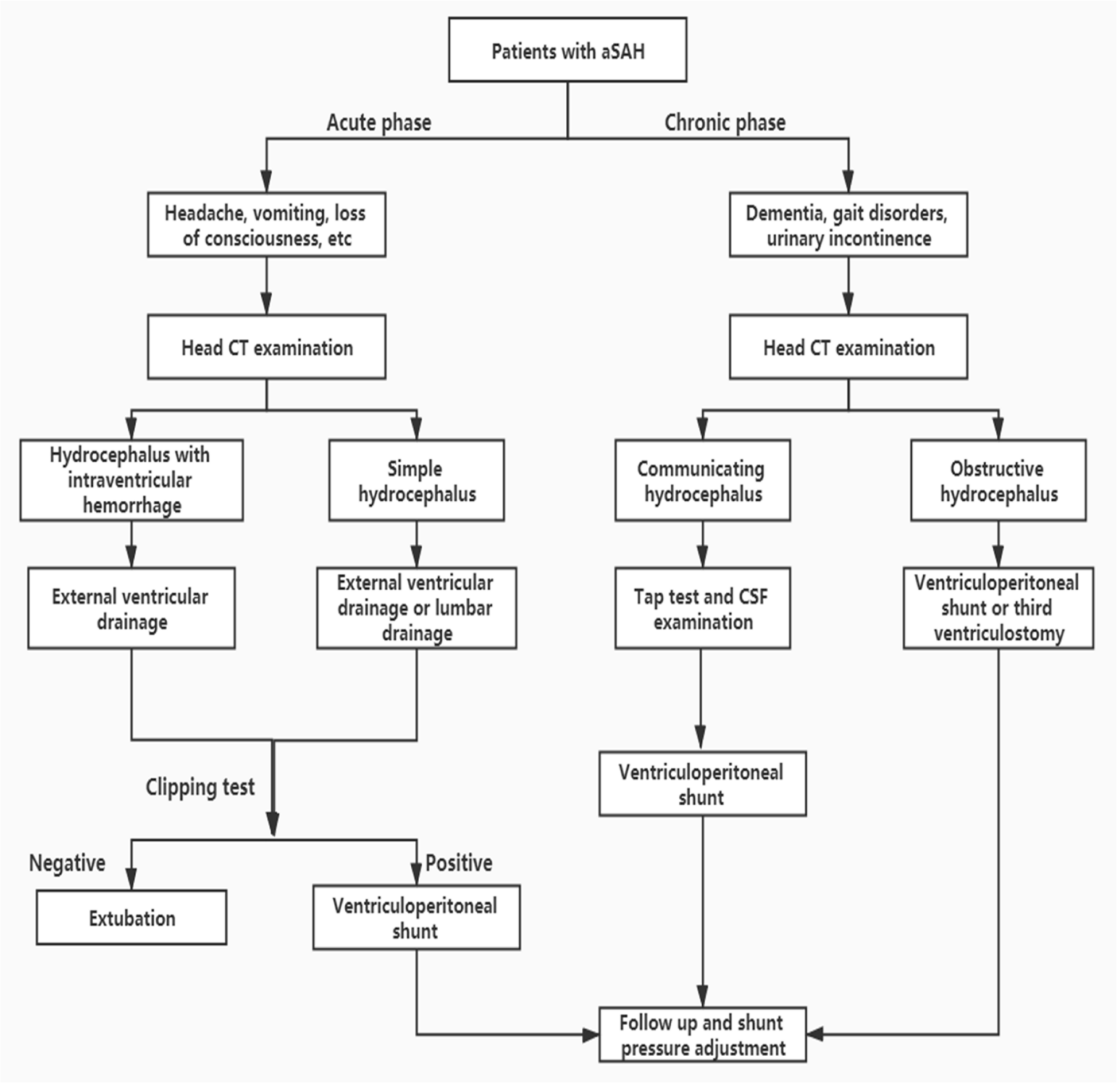
Flow chart of management of aSAH

The main purpose of treatment is to release high intracranial pressure or to tackle with pathological changes of brain tissue caused by hydrocephalus so as to prevent further damage of neurological function. The principle of treatment is to investigate the cause of hydrocephalus, select appropriate therapy, and manage ventricular enlargement [25]. For patients of hydrocephalus with no obvious clinical symptoms, conservative treatment and close clinical observation is preferred. Some patients, especially acute hydrocephalus ones, may demonstrate an arrested hydrocephalus or even gradually relieved by themselves. However, for patients with clinically aggravated consciousness disorder or neurological condition improved once but worsened later, and typical image features of hydrocephalus and progressively aggravated ones, treatment is needed in time (Fig. 4).

### Medical treatment

These are medication that inhibits CSF secretion, osmotic dehydrating agents, and diuretics that reduce intracranial pressure. Medical treatment is usually recommended for temporary relieve or adjuvant treatment of aSAH-H.

### Surgical treatment

#### CSF drainage

To release certain volume of CSF through surgical procedure such as intermittent lumbar puncture, controlled lumbar drainage, external ventricular drainage and subcutaneous Ommaya Reservoir Placement, so as to temporarily relieve intracranial hypertension and drain bloody CSF [25]. When it is difficult to separate the brain tissue due to hydrocephalus, the lateral ventricle drainage can be performed through the operation area and left as needed, which can be removed at the end of the operation or several days after the operation. For patients with acute hydrocephalus, especially with ventricular hemorrhage, external ventricular drainage is usually adopted. If the third and fourth ventricles are unobstructed, continuous lumbar drainage can also be applied [26]. In the acute stage, due to the high protein concentration in CSF and the poor tolerance of patients, it has higher risk of complications performing ventriculoperitoneal shunt. For patients undergoing craniotomy, subarachnoid drainage can be placed through lumbar puncture before operation, so as to continuously drain the CSF after operation or to perform fenestration of lamina terminalis during the operation, which are all beneficial to CSF circulation.

External ventricular drainage usually choose the angulus frontalis for puncture, a position 2 cm in front of the coronal suture, 2 cm beside the median line, and point to the connection of the double external acoustic meatus. Place the catheter at a depth of 5–8 cm. The right angulus frontalis of the nondominant hemisphere is preferred for puncture. If the left lateral ventricle is significantly enlarged with hemorrhage, the left angulus frontalis can be selected for puncture, and the drainage tube advances in the subcutaneous tunnel for about 5 cm, in order to reduce the possibility of retrograde infection. Placing ventricular drainage before managing aneurysm would put patients at great risk, because fluctuation of intracranial pressure increases the risk of aneurysm rupture. Therefore, aneurysm should be managed earlier, and after the aneurysm is successfully treated, the ventricular drainage is able to drain bloody CSF, thus promoting the circulation and absorption of CSF [27–29]. The timing of clipping the drainage tube is particularly important for patients with CSF drainage. The drainage tube should be clipped as soon as possible according to the condition. If the patient has vasospasm, the timing of clipping should be postponed [29, 30]. The volume of drainage can be gradually reduced while closely monitoring intracranial pressure and finally to clip and remove the drainage [27]. After the drainage tube is clipped, intracranial pressure, consciousness, headache, and other indicators should be closely observed. If clinical symptoms of the patient do not deteriorate within 24 h, and head CT does not show continuous ventricular enlargement, the clipping is satisfactory, and it can be removed [27, 29]. If intracranial pressure is ≥ 25 cm H_2_O for more than 5 min after clipping, the drainage tube should be unclipped immediately, and the clipping test failed [29]. The next day, clipping test can be performed again. If the clipping test fails for more than 3 times, it may need shunt procedure [28]. If intracranial pressure cannot be monitored in real time during clipping, we should have a close eye on clinical manifestations like headache, consciousness, and pupil changes.

### CSF shunt surgery

It includes ventriculoperitoneal shunt, ventriculoatrial shunt, lumbar-peritoneal shunt, etc. Ventriculoperitoneal shunt is mostly used for communicating hydrocephalus [2, 9]. Although ventriculoatrial shunt is less performed, it is still an indispensable choice for patients with abdominal infection.

In clinical practice, the treatment principles of acute and chronic hydrocephalus are different. In the acute phase, due to the high protein concentration of CSF and the poor tolerance of patients, there are more complications if ventriculoperitoneal shunt is performed, so priority should be external ventricular drainage or lumbar drainage. Ventriculoperitoneal shunt can be applied if the patient is as follows: shunt-dependent after treatment, the drainage tube cannot be removed, protein concentration of CSF is significantly reduced, and there is no intracranial infection.

Most of the chronic hydrocephalus patients fall in category of communicating hydrocephalus. In this case, ventriculoperitoneal shunt is usually preferred [4]. Lumboperitoneal shunt also applies for communicating hydrocephalus with its advantage of avoiding ventricular injury, ventricular collapse-induced shunt obstruction, and secondary infection, but there is a risk of secondary cerebral hernia. In order to ensure the effectiveness of the treatment, high-volume lumbar puncture TAP test or continuous lumbar drainage is needed. In a lumbar puncture wherein a large volume (typically 40–50 mL) of CSF is removed, gait testing, patient’s response, and consciousness are observed. Transient recovery in gait, conscious, and patient’s response after the TAP has been considered a positive prognostic indicator for surgery [31]. However, some patients with negative TAP test also benefit a lot in terms of symptoms from shunt procedure, which tells the story of unreliability of TAP test sometime. Continuous lumbar drainage, 150–200 mL per day, > 3 days, is necessary, to observe whether clinical symptoms have been improved, so as to predict the effectiveness of ventricular-peritoneal shunt more accurately. Surgical indications are as follows: chronic hydrocephalus, unimproved or deteriorated neurological symptoms in case of enlarged ventricular system, TAP test positive, always with bilateral angulus frontalis interstitial edema, and disappearance of cortical sulci. Contraindications of CSF shunt were as follows: acute intracranial hemorrhage, uncontrolled intracranial infection;,infection foci in the shunt path, and peritoneal infection. Although the low-pressure shunt valve is more efficient, in order to minimize the occurrence of subdural hematoma, it is recommended to use a medium-pressure shunt valve (pressure range 90–110 mm H_2_O), and the specific parameter needs to be adjusted according to the TAP results.

Site of ventricle puncture depends on specific patient and doctor’s clinical judgement. Angulus frontalis, cornu occipitale, or pars triangularis is the candidate site. In recent years, intraoperative navigation has been used in intraventricular puncture to level-up success rate of puncture and avoid multi-puncture injury. For inexperienced doctors or patients with complicated conditions, intraoperative navigation is recommended [32]. Peritoneal end of shunt catheter is usually implanted at inverse McBurney point and on facies diaphragmatica hepatis. Application of laparoscopy makes procedure easier and safer.

#### CSF intracranial diversion

Endoscopic third ventriculostomy (ETV) represents a popular procedure for obstructive hydrocephalus preferring CSF intracranial diversion [33], followed by lamina terminalis fenestration, endoscopic aqueductoplasty, pellucid septostomy, and intraventricular neomembrane ostomy.

Infratentorial intracisternal obstructive hydrocephalus refers to the communication between ventricles, and subarachnoid space/cerebellomedullary cistern is unobstructed; however, the communication between cerebellomedullary cistern and prepontine cistern (pontine cistern) is obstructed, so the CSF cannot flow to supratentorial to be reabsorbed. This kind of hydrocephalus lacks direct image signs, and the indirect signs mainly manifested as the protruding bottom of the third ventricle downward and the lamina terminalis protruding forward. The third ventriculostomy can establish a communication between ventricular system and subarachnoid space, which is conducive to the circulation and absorption of CSF.

#### Recommendations

For acute hydrocephalus, external ventricular drainage is recommended, and clipping test helps to remove the drainage tube safely; for chronic hydrocephalus, most of which are communicative hydrocephalus, ventriculoperitoneal shunt is recommended, and laparoscopy is recommended for abdominal catheterization; TAP test is required before operation, and a positive result indicates effectiveness of ventriculoperitoneal shunt.

## Postoperative complications and management

### Complications and management of ventricular drainage

Complications of external ventricular drainage mainly include intracerebral hemorrhage and intracranial infection [34, 35]. Intracerebral hemorrhage includes ventricular hemorrhage, intraparenchymal hemorrhage, and subdural hematoma. The first and second hemorrhage is mainly due to puncture injury and repeated punctures, and coagulation dysfunction is also an important contributing factor [35]. So accurate positioning is the key to reduce bleeding from puncture injury. Surgical procedure is required if large volume of bleeding occurs. Subdural hematoma is mainly due to cortical vascular damage of sudden decrease of intracranial pressure. Therefore, adjusting the pressure before surgery and maintaining optimal shunt pressure are the key to prevent subdural hematoma. The speed and volume of CSF drainage must be accurately controlled. Excessive drainage can have the risk of bleeding, headache, etc. An important risk factor for intracranial infection is the duration of placement of drainage tube. The longer its placement, the higher the risk of infection. Subcutaneous tunnel and earlier extubation are important methods to reduce risks of intracranial infections [34, 36].

### Complications and management of ventriculoperitoneal shunt

The incidence of complications after ventriculoperitoneal shunt is relatively high, about 20% perioperatively, including intracranial hemorrhage, infection, shunt obstruction, underdrainage, or over-drainage of shunt [15, 37, 38]. Intracranial hemorrhage is a serious complication after ventriculoperitoneal shunt. According to the location of occurrence, it can be divided into intracerebral hematoma and subdural hematoma, or according to the time of occurrence, it can be divided into acute hematoma and delayed hematoma [39]. Intracerebral hematoma is mainly caused by vascular injury during puncture, for repeated puncture is closely related to intracerebral hemorrhage, as well as coagulation dysfunction [39]. The key of prevention is gentle operation, accurate location, and avoidance of repeated puncture. Subdural hematoma is mainly caused by intracranial pressure change after shunt operation; the collapsed brain tissue retracts and tears cortical vein, resulting in subdural hematoma [39, 40]. According to TAP test, the initial pressure of the shunt can be adjusted to the level of actual or slightly lower than the intracranial pressure and make adjustment later.

Infection is the main complication after shunt operation which usually occurs within 1 month. The incidence is about 2.5–7.0%. It mainly includes intracranial infection, subcutaneous tunnel infection of shunt catheter, and peritonitis, among which intracranial infection is the most serious and prognosis the worst [37, 38]. The shunt operation must be performed in laminar airflow theater, and replacement of gloves should be strictly observed when implanting the catheter, and limitation of entry and exit as well as medics in the theater is applied. Antibiotic-impregnated shunt catheter is used when necessary. The postoperative infection of ventriculoperitoneal shunt is caused by multiple factors and procedures. We should emphasize the concept of surgical asepsis and prevent postoperative infection. Patients with mild intracranial infection can be treated with combined antibiotic therapy, while patients with severe infection need to remove the infected shunt tube, and external ventricular drainage should be performed. Only when the infection is controlled, i.e., continuous culture of CSF is negative, CSF leukocyte count < 30, glucose ratio (CSF glucose/serum glucose) < 0.4, and CSF protein < 0.5 g/L, can the shunt be performed again.

**Fig. 5:**
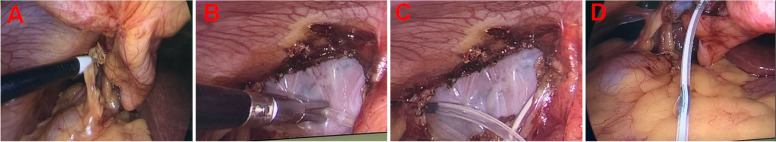
Release of distal end occlusion by laparoscopy. **A**–**C** Shunt catheter blocked by omentum. **D** Shunt catheter after dissection

Shunt occlusion includes proximal (ventricular catheter) and distal (peritoneal catheter) occlusion. One important cause of proximal occlusion is the high concentration of protein in CSF. The ventricular catheter may become obstructed by debris, coagulum, or contact with choroid plexus or improper implant of catheter. At present, most scholars believe that the ideal position of proximal catheter is free in the angulus frontalis of lateral ventricle, flush with Moro foramen, disconnecting ventricular wall, and away from choroid plexus. This requires high precision, which intraoperative navigation can help [32]. In addition, lumbar-peritoneal shunt can avoid proximal catheter occlusion due to ventricular retraction. Pulling out the ventricular end to clean up the blocked material remains controversial when ventricular end of the shunt is blocked, because there is a high risk of intracerebral bleeding. The distal end may become blocked by omentum or peritoneal adhesions. Shunt valve has good resilience and compliance, but improvement cannot be made after lowering pressure; this is a strong indicator for distal catheter occlusion. Laparoscopy can be adopted to release distal occlusion satisfactorily (Fig. 5).

Symptoms of over-drainage include low ICP headache, acute subdural hematoma, chronic subdural effusion, and slit ventricle syndrome [40]. Before the anti-siphon device was widely used, the siphon effect induced by positional changes was an important cause for over-drainage. Now the anti-siphon device is widely used clinically, and low pressure of the shunt catheter is the main cause for over-drainage. For underdrainaged patients, the clinical manifestations and image test show no obvious improvement after ventriculoperitoneal shunt. The reason for underdrainage is mainly due to high pressure of shunt catheter or occlusion or mechanical failure. It is important to adjust initial pressure of the shunt, and reference can be made to TAP test result before surgery. Optimal pressure is achieved with reference to clinical manifestation and image results at a later time.

#### Recommendation

High incidence rate of complication perioperatively is for ventriculoperitoneal shunt. Strict aseptic procedures should be observed during operation. Anti-siphon and pressure-adjustable shunt catheter is recommended, and adjustment should be made according to clinical manifestations, image results, and laboratory examinations. There is good resilience and compliance of the shunt valve, but LP pressure much higher than valve pressure indicates distal end occlusion.

## Conclusions

Hydrocephalus is a common complication following aSAH. External ventricular drainage is recommended for acute hydrocephalus. For chronic hydrocephalus, if it is communicating hydrocephalus, ventriculoperitoneal shunt is recommended; if it is obstructive hydrocephalus, ventriculoperitoneal shunt or third ventriculostomy is recommended. Ventriculoperitoneal shunt is an important therapy for communicating hydrocephalus but with more complications. Therefore, surgical indications and protocols must be observed strictly.

This expert consensus is for adults only. It serves as a reference for clinicians in clinical diagnosis and treatment. It will not be interpreted in any sense and has any legal effect. More research is expected in China in this field, and the views in this consensus will be refined with the progress of diagnosis and treatment techniques and experimental research.
